# Epinephrine: The Drug of Choice for Anaphylaxis--A Statement of the World Allergy Organization

**DOI:** 10.1097/1939-4551-1-S2-S18

**Published:** 2008-07-15

**Authors:** Stephen F Kemp, Richard F Lockey, F Estelle R Simons

**Affiliations:** 1 Chairman (USA; 2 (USA; 3 (Canada

**Keywords:** anaphylaxis, epinephrine, management, prevention

## Abstract

Anaphylaxis is an acute and potentially lethal multisystem allergic reaction. Most consensus guidelines for the past 30 years have held that epinephrine is the drug of choice and the first drug that should be administered in acute anaphylaxis. Some state that properly administered epinephrine has no absolute contraindication in this clinical setting. A committee of anaphylaxis experts assembled by the World Allergy Organization has examined the evidence from the medical literature concerning the appropriate use of epinephrine for anaphylaxis. The committee strongly believes that epinephrine is currently underused and often dosed suboptimally to treat anaphylaxis, is underprescribed for potential future self-administration, that most of the reasons proposed to withhold its clinical use are flawed, and that the therapeutic benefits of epinephrine exceed the risk when given in appropriate intramuscular doses.

## 

Epinephrine is the treatment of choice and the first drug administered for acute anaphylaxis, as confirmed internationally by most consensus anaphylaxis guidelines published in the English language over the past 30 years[1-17]. Therapeutic recommendations for epinephrine use in anaphylaxis are largely based on clinical pharmacology studies, clinical observation, and animal models.

Anaphylaxis often occurs outside of a medical setting, for example, after food ingestion or an insect sting, and the onset may be sudden and without warning. Severity varies from episode to episode even with an identical stimulus in the same patient. Recognition and diagnosis of anaphylaxis is sometimes difficult for health care professionals and for individuals without medical training[18].

Few controlled clinical trials, and no placebo-controlled trials, have been performed in anaphylaxis because of the nature of the disease[19]. Randomization to a nonepinephrine treatment would be unethical because of the preponderance of data showing that expeditious treatment with epinephrine is optimal, if not critical, for survival in many instances[20-25]. The following discussion reviews current evidence for the use of epinephrine in anaphylaxis.

## Definition

The traditional nomenclature for anaphylaxis reserves the term *anaphylactic *for immunoglobulin E (IgE)-dependent reactions and the term *anaphylactoid *for IgE-independent events, which are clinically indistinguishable. The World Allergy Organization, a worldwide federation of national and regional allergy and clinical immunology societies and organizations dedicated to raising awareness and advancing excellence in clinical care, education, research, and training in allergy and clinical immunology, recommends that this terminology be replaced with *immunologic *(IgE-mediated and non-IgE-mediated [eg, IgG and immune complex complement-mediated]) and *nonimmunologic *anaphylaxis[26]. Therefore, in this article, the term *anaphylaxis *refers to both immunologic and nonimmunologic anaphylaxis.

## Methods

**Figure 1:**
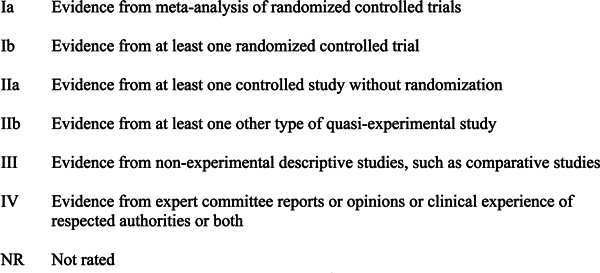
**Categorization of evidence**. Adapted from Shekelle et al.[27]

A literature search of Medline (1966 to present) was conducted using the key words *anaphylaxis *and *epinephrine *and articles from the personal anaphylaxis file collections of the authors were also included. Cross-references were accessed when deemed appropriate. References have been categorized by degree of evidence, where possible[27] (Figure 1).

## Anaphylaxis in Perspective

Anaphylaxis is an acute and potentially lethal multi-system allergic reaction in which some or all of the following signs and symptoms occur: diffuse erythema, pruritus, urticaria and/or angioedema; bronchospasm; laryngeal edema; hypotension; cardiac arrhythmias; feeling of impending doom; unconsciousness and shock. Other earlier or concomitant signs and symptoms can include itchy nose, eyes, pharynx, genitalia, palms, and soles; rhinorrhea; change in voice; metallic taste; nausea, vomiting, diarrhea, abdominal cramps, and bloating; lightheadedness; headache; uterine cramps; and generalized warmth.

The US National Institute of Allergy and Infectious Diseases (Bethesda, Md) and the Food Allergy and Anaphylaxis Network (Chantilly, Va) convened symposia in 2004 and 2005, during which an international and interdisciplinary group of representatives and experts from 16 professional, government, and lay organizations attempted, among other tasks, to establish clinical criteria that would increase diagnostic precision in anaphylaxis[16]. The working definition proposed is the following: "Anaphylaxis is a serious allergic reaction that is rapid in onset and may cause death." The group proposed that anaphylaxis is likely to be present clinically if any one of 3 criteria is satisfied within minutes to hours: (1) acute onset of illness with involvement of skin, mucosal surface, or both, and at least one of the following: respiratory compromise, hypotension, or end-organ dysfunction; (2) 2 or more of the following occur rapidly after exposure to a likely allergen: involvement of skin or mucosal surface, respiratory compromise, hypotension, or persistent gastrointestinal symptoms; and (3) hypotension develops after exposure to a known allergen for that patient: age-specific low blood pressure or decline of systolic blood pressure of greater than 30% compared with baseline[16]. The group concluded that these criteria "are likely to capture more than 95% of cases of anaphylaxis." The implication from this definition could be interpreted to mean that more than just cutaneous and other even less severe symptoms need to be present before epinephrine is administered. However, the Anaphylaxis Working Group report also states that, "There undoubtedly will be patients who present with symptoms not yet fulfilling the criteria of anaphylaxis yet in whom it would be appropriate to initiate therapy with epinephrine, such as a patient with a history of near-fatal anaphylaxis to peanut who ingested peanut and within minutes is experiencing urticaria and generalized flushing."

In summary, anaphylaxis occurs as part of a clinical continuum. It can begin with relatively minor symptoms and rapidly progress to a life-threatening respiratory and cardiovascular reaction. Delaying treatment until the development of multiorgan symptoms, as under the clinical criteria for diagnosis by the Anaphylaxis Working Group report, may be risky because the ultimate severity of anaphylaxis is difficult or impossible to predict at the time of onset of the episode. Therefore, some of the authors and members of the World Allergy Organization Ad Hoc Committee on Epinephrine and Anaphylaxis recommend that any symptoms of anaphylaxis, such as generalized pruritus, erythema, urticaria, and angioedema alone, and any other systemic symptom including those not involving vital organs, should be treated immediately and as necessary with appropriate intramuscular doses of epinephrine in an attempt to prevent more severe anaphylaxis from occurring.

Conversely, symptoms clearly attributable to another diagnosis for which the clinical probability is much higher, for example, generalized pruritus, urticaria, and angioedema associated with new-onset acute urticaria and/or angioedema, or with an exacerbation of chronic urticaria and/or angioedema, do not necessarily have to be treated with epinephrine.

Thus, there are 2 schools of thought as to when epinephrine should be given intramuscularly for anaphylaxis or what appear to be early symptoms of anaphylaxis. One recommends that epinephrine should be given as described, by the US National Institute of Allergy and Infectious Diseases and the Food Allergy and Anaphylaxis Network,[16] whereas another group would go even further and recommend that epinephrine should be administered as early as possible after the onset of the least serious or minor symptoms, particularly when the offending agent or allergen is administered parenterally. Evidence demonstrates that parenteral delivery of the offending allergen or causative agent is associated with more rapid absorption and potentially catastrophic anaphylaxis than the oral route of administration. However, any route of administration, oral or parenteral, can cause anaphylaxis and begin with minor symptoms and result in anaphylactic death.

Foods, medications, insect stings, and allergen immunotherapy injections are the most common provoking factors for anaphylaxis, but it can be induced by any agent capable of producing a sudden degranulation of mast cells or basophils[28]. Anaphylaxis caused by diagnostic and therapeutic interventions is almost unavoidable in medical practice and occurs in a variety of clinical scenarios[16]. The lifetime individual risk of anaphylaxis is presumed to be 1% to 3%, with a mortality rate of 1%,[28] and the prevalence of anaphylaxis may be increasing[29]. Therefore, all physicians must be able to recognize anaphylaxis, treat it appropriately, and provide recommendations to prevent future episodes.

Signs and symptoms of anaphylaxis vary, but cutaneous features (generalized erythema, pruritus, urticaria, and angioedema) are the most common overall[28]. Reactions may be immediate and uniphasic, or they may be delayed in onset, biphasic (recurrent), or protracted. Biphasic anaphylaxis occurs in 1% to 20% of anaphylaxis, and symptoms may recur 1 hour to 72 hours (most within 8 hours) after apparent resolution of the initial phase[30]. The severity of the initial phase of an anaphylactic reaction is not predictive of either biphasic or protracted anaphylaxis, although failure to give an adequate dose of epinephrine initially may be associated with increased risk of biphasic anaphylaxis. Monitoring of patients for 24 hours or more after apparent recovery from the initial phase may be necessary in more severe cases because life-threatening manifestations of anaphylaxis may recur. Data are limited concerning the frequency with which 2 or more doses of epinephrine are needed to treat anaphylaxis (reports range from 16% to 36%), and multiple cofactors may be involved[31-33].

Respiratory compromise and cardiovascular collapse cause most fatalities[28,34]. An analysis of 202 anaphylaxis fatalities occurring in the United Kingdom from 1992 to 2001 ascertained that the interval between initial onset of food anaphylaxis symptoms and fatal cardiopulmonary arrest averaged 25 to 35 minutes, which was longer than for insect stings (10-15 minutes) or for drugs (mean, 5 minutes in hospital; 10-20 minutes prehospital)[34].

Increased vascular permeability during anaphylaxis can shift up to 35% of intravascular fluid to the extravascular space within 10 minutes[35]. The intrinsic compensatory response to anaphylaxis (endogenous epinephrine and other catecholamines, as well as angiotensin II, endothelin-1, etc) also influences the extent of clinical manifestations and, when adequate, may be lifesaving independent of medical intervention, which sometimes contributes to diagnostic and therapeutic confusion. Because mast cells accumulate at sites of coronary atherosclerotic plaques and IgE antibodies bound to mast cells can trigger mast cell degranulation, some investigators have suggested that anaphylaxis may lead to myocardial ischemia by promoting plaque rupture[36,37]. Stimulation of the H_1 _histamine receptor may also produce coronary artery vasospasm[37,38].

## Pharmacology of Epinephrine

**Figure 2:**
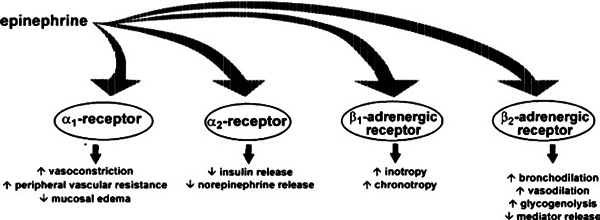
**Adrenergic effects of epinephrine**. Adapted from Simons[40].

The pharmacology of epinephrine is reviewed in detail elsewhere (Figure 2)[39,40]. At recommended dosages and routes of administration, the α-adrenergic vasoconstrictive effects reverse peripheral vasodilation, which alleviates hypotension and also reduces erythema, urticaria, and angioedema. Local injection of epinephrine may also minimize further absorption of antigen from a sting or injection, but this has not been studied systematically. The β-adrenergic properties of epinephrine cause bronchodilation, increase myocardial output and contractility, and suppress further mediator release from mast cells and basophils[41,42]. Epinephrine administered in low concentrations (eg, 0.1 μg/kg) paradoxically can produce vasodilation, hypotension, and increased release of inflammatory mediators[39,43].

Epinephrine administration enhances coronary blood flow. Two mechanisms are probably responsible: an increased duration of diastole compared with systole and a vasodilator effect caused by increased myocardial contractility. These actions usually offset the vasoconstrictor effects of epinephrine on the coronary arteries[39,44].

Rapid achievement of peak plasma and tissue epinephrine levels seems to optimize survival because retrospective human studies demonstrate that delayed administration is associated with poor outcomes[20,21]. However, epinephrine administration during anaphylaxis is not always effective, and patients may still die[20-25]. Reasons may be multifactorial and include delayed administration, inadequate doses, inappropriate route of administration, use of epinephrine that has passed its expiration date, leading to inadvertent administration of an inadequate dose, or an underlying disease, such as poorly controlled asthma, cardiovascular disease, mastocytosis, and perhaps other serious systemic disorders[40,45]. A study done in a canine model also demonstrates that achievement of peak epinephrine plasma levels and hemodynamic recovery is not as effective when epinephrine administration is delayed until hypotension has developed[46].

**Figure 3:**
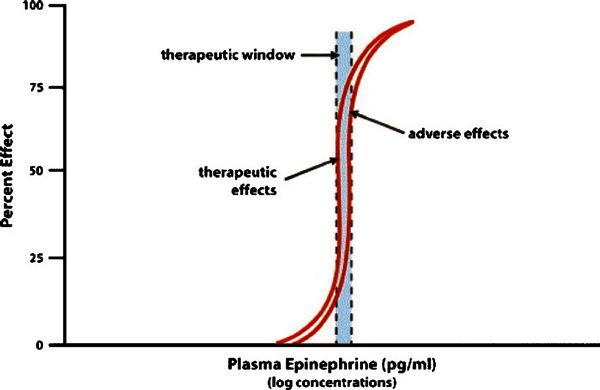
**Therapeutic window of epinephrine**. Adapted from Simons[18].

Epinephrine has a relatively narrow therapeutic window (relative benefit vs risk; Figure 3). Common pharmacological effects that occur at recommended doses via any route of administration include agitation, anxiety, tremulousness, headache, dizziness, pallor, or palpitations[39]. Rarely, and usually associated with overdosage or overly rapid rate of intravenous infusion, epinephrine administration might contribute to or cause myocardial ischemia or infarction,[47-52] pulmonary edema,[53,54] prolonged QTc (QTc = QT interval divided by the square root of the RR interval [in seconds] of the electrocardiogram) interval,[55] ventricular arrhythmias, accelerated hypertension, and intracranial hemorrhage in adults and children alike[41,56]. Nonetheless, some patients have survived massive overdoses of epinephrine, with no evidence of myocardial ischemia[57,58]. Particularly vulnerable populations are those individuals at the extremes of age and those with hypertension, peripheral vascular disease, ischemic heart disease, or untreated hyperthyroidism (increased number of β-adrenergic receptors in the vasculature of these individuals render the myocardium more sensitive to β-adrenergic effects of epinephrine)[59]. Certain medications might also increase the risk of adverse events from drug interactions[13,18,42,59]. Some medications decrease the effectiveness of endogenous catecholamine stores or exogenously administered epinephrine (β-adrenergic blockers), interfere with intrinsic compensatory responses to hypotension (angiotensin-converting enzyme inhibitors and possibly angiotensin II receptor blockers), or impede epinephrine metabolism and lead to increased plasma and tissue concentrations (tricyclic antidepressants and monoamine oxidase inhibitors). The β-adrenergic antagonists and α-adrenergic antagonists can also potentially exaggerate pharmacological effects of epinephrine by permitting unopposed α-adrenergic (vasoconstrictor) and β-adrenergic (vasodilator) effects, respectively. Cocaine and amphetamines sensitize the myocardium to effects of epinephrine, thus increasing the risk of toxicity.

However, none of these circumstances pose an absolute contraindication to epinephrine administration for anaphylaxis[13].

## Management of Anaphylaxis

Physician and other health care professionals who perform procedures or administer medications should have available the basic therapeutic agents used to treat anaphylaxis [4,7,13] : (1) stethoscope and sphygmomanometer; (2) tourniquets, syringes, hypodermic needles, large-bore needles (eg, 14- or 16-gauge); (3) injectable aqueous epinephrine 1:1000 (1 mg in 1 mL; physicians are being urged to express doses in mass concentration, eg, 1 mg in mL, rather than as ratios, eg, 1:1000, which have been identified as a source of dosing errors with epinephrine and other medications); (4) equipment and supplies for administering supplemental oxygen; (5) equipment and supplies for administering intravenous fluids; (6) oral or laryngeal mask airway; (7) diphenhydramine or similar injectable antihistamine; (8) ranitidine or other injectable H2 antihistamine; (9) corticosteroids for intravenous injection; and (10) vasopressors (eg, dopamine or norepinephrine). Glucagon, an automatic defibrillator, and 1-way valve face mask with oxygen inlet port are other supplies that some clinicians might find desirable depending on the individual clinical setting[13].

Assessment and maintenance of airway, breathing, circulation, and mentation are necessary before proceeding to other management steps. Patients are monitored continuously to facilitate prompt detection of any clinical changes or treatment complications. Placement of a patient in the recumbent position with elevation of the lower extremities is strongly recommended because management in the sitting or upright position has contributed to poor outcomes in some patients[34].

### When to Administer Epinephrine

Epinephrine should be administered simultaneously with the above measures[12-14]. By expert consensus based on anecdotal evidence, there is no absolute contraindication to epinephrine administration in anaphylaxis[13]. It can be administered in doses appropriate for the severity of the reaction, regardless of the initial signs and symptoms of anaphylaxis. All subsequent therapeutic interventions depend on the initial response to epinephrine. Development of toxicity or inadequate response to epinephrine injections indicates that additional therapeutic modalities are necessary[13]. Table 1 outlines a sequential approach to anaphylaxis treatment. Modalities used in concert with epinephrine are reviewed in detail elsewhere[10-14].

**Table 1:** Management of Acute Anaphylaxis

I. Immediate intervention
a. Assessment of airway, breathing, circulation, and adequacy of mentation
b. Administer epinephrine intramuscularly every 5 to 15 minutes, in appropriate doses, as necessary, depending on the presenting signs and symptoms of anaphylaxis, to control signs and symptoms and prevent progression to more severe symptoms, such as respiratory distress, hypotension, shock, and unconsciousness.
II. Possibly appropriate subsequent measures depending on response to epinephrine
a. Place patient in recumbent position and elevate lower extremities
b. Establish and maintain airway
c. Administer oxygen
d. Establish venous access
e. Isotonic sodium chloride solution intravenously for fluid replacement
III. Specific measures to consider after epinephrine injections, where appropriate
a. Consider epinephrine infusion
b. Consider H_1 _and H_2 _antihistamines
c. Consider nebulized β_2 _agonist (eg, albuterol [salbutamol]) for bronchospasm resistant to epinephrine
d. Consider systemic corticosteroids
e. Consider vasopressor (eg, dopamine)
f. Consider glucagon for patient taking β-blocker
g. Consider atropine for symptomatic bradycardia
h. Consider transportation to an emergency department or an intensive care facility
i. For cardiopulmonary arrest during anaphylaxis, high-dose epinephrine and prolonged resuscitation efforts are encouraged, if necessary (see reference for specific details)

Adapted from Lieberman et al.[13]

### Epinephrine Injections

Expert consensus and anecdotal evidence indicate aqueous epinephrine 1:1000 dilution (1 mg in 1 mL), 0.2 to 0.5 mg (0.01 mg/kg in children; maximum dose, 0.3 mg) administered intramuscularly every 5 to 15 minutes or as necessary, depending on the severity of the anaphylaxis, should be used to control symptoms and sustain or increase blood pressure[12-14]. Efficacy comparisons of intramuscular injections to subcutaneous injections have not been done during acute anaphylaxis. However, absorption is complete and more rapid and plasma levels are higher in asymptomatic adults and children who receive epinephrine intramuscularly in the anterolateral thigh (vastus lateralis)[60,61]. In overweight and obese individuals, the thickness of the subcutaneous fat pad may preclude intramuscular access[62-64]. Table 2 provides some examples of clinical scenarios where the pros and cons of epinephrine use might be weighed[65,66].

**Table 2:** Clinical Scenarios for Epinephrine Use Outside of a Medical Facility

For Discussion Purposes	
**Clinical Findings**	**Use of Epinephrine?**

Generalized urticaria develops in a 28-yr-old fire ant-allergic individual stung by ant while playing in the yard. Currently receives ant immunotherapy based on positive skin test response to fire ant whole body extract but is not yet at maintenance dosage (6 wk of therapy on conventional\buildup schedule).	Pro: inject immediately; past anaphylaxis and current findings away from medical facility Con: do not inject immediately; wait for symptoms involving another organ system
A 45-yr-old yellow jacket-allergic farmer has just been stung after disturbing nest with tractor. History of hypotension and rapid syncope in past stings. Currently receives venom immunotherapy but is not yet at maintenance (last dose was 1 mL [L]). No current symptoms.	Pro: inject immediately in view of past severe anaphylaxis; low risk of serious side effects from injected epinephrine; some risk of severe symptoms because he has not reached maintenance Con: do not inject immediately; wait for symptoms
A 17-yr-old individual develops paroxysmal sneezing within 5 min of receiving allergen immunotherapy injection	Pro: inject immediately; rapid onset of symptoms may be associated with severe anaphylaxis; low risk of serious side effects from injected epinephrine; antihistamines are second-line agents in anaphylaxis Con: do not inject immediately; wait for other symptoms if suspect sneezing could be due to transient respiratory irritant exposure or seasonal allergy exacerbation if it occurs during pollen season of a pollen-allergic individual.
A 7-yr-old child with mild persistent asthma and clinical history of peanut allergy (wheeze, hives that "get better after vomiting") experiences sudden cough and wheeze while playing outside 15 min after eating a cookie in school cafeteria; has no other symptoms; has albuterol metered-dose inhaler and epinephrine autoinjector available	Pro: inject immediately; history is strongly suggestive of past anaphylaxis; safety of cookie is uncertain; signs and severity of anaphylaxis can vary from episode to episode in the same individual; delayed treatment or treating anaphylaxis with salbutamol (albuterol) alone could have adverse outcome; low risk of serious side effects from injected epinephrine Con: do not inject immediately; for possible asthma (eg, exercise-induced or pollen exposure), assess response to salbutamol first.

Anaphylaxis occurs as part of a continuum, and delaying treatment until multiorgan dysfunction is present is risky. The recommendations in this table apply regardless of comorbid conditions because there is no absolute contraindication to epinephrine administration during anaphylaxis. Physicians and other health care professionals should instruct patients at risk for anaphylaxis outside of a medical facility to err on the side of caution and self-administer epinephrine if there is any doubt anaphylaxis is either present or imminent.

Adapted from Sicherer and Simons[65].

Epinephrine autoinjectors, which are easy to use and will inject through clothing, are currently available in 2 fixed doses: 0.15 mg and 0.3 mg. The potential exists for overdosage in infants receiving the 0.15 mg, overdosage in some small children receiving the 0.3 mg dose, and for underdosage in many adolescents receiving the 0.15 mg dose[17,40]. The relative benefits and risks of dosage might vary with each individual, but autoinjectors with 0.15 mg of epinephrine are recommended for otherwise healthy children who weigh 10 to 25 kg (22-55 lb) and autoinjectors with 0.3 mg of epinephrine for children who weigh approximately 25 kg (55 lb) or more[17,40]. Providing parents with an epinephrine ampule, syringe, and needle is not an appropriate option unless autoinjectors are not available for prescription[66].

### Intravenous Epinephrine

Epinephrine (1:10,000 or 1:100,000 dilutions) should be administered by infusion during cardiac arrest or to unresponsive or severely hypotensive patients who have failed to respond to intravenous volume replacement and several epinephrine injections[13]. One group of investigators suggest that the early use of intravenous epinephrine is safe, effective, and well tolerated when the rate is titrated to clinical response, but this has not been evaluated systematically in a cohort study comparing this modality to epinephrine intramuscular injections[67].

### Inhaled Epinephrine

Some physicians recommend inhalation of epinephrine as an alternative to injection during anaphylaxis, but perioral paresthesias, bad taste, and gastrointestinal effects are dose-limiting, and it may not achieve prompt significant increases in plasma epinephrine concentrations[68,69]. No direct comparisons have been made between the inhaled and the intramuscular routes of epinephrine administration.

## Follow-Up and Observation after Anaphylaxis

Observation periods should be individualized and based on such factors as comorbid conditions and distance from the patient's home to the closest emergency facility, particularly because there are no reliable predictors of biphasic anaphylaxis[13]. After resolution of the acute episode, patients should be discharged with an epinephrine autoinjector and properly instructed on how to self-administer it in case of a subsequent episode. They should receive an individualized Anaphylaxis Emergency Action Plan[18]. Patients should also have ready access to emergency medical services to facilitate prompt transportation to the closest emergency department (ED) for treatment after injecting the additional epinephrine.

## Use of Epinephrine by Health Care Professionals

Numerous guidelines on anaphylaxis have been published, but physicians and other health care professionals often do not follow them. For example, investigators determined by questionnaire that only 4 (5%) of 78 senior house officers beginning ED responsibilities in the United Kingdom would administer epinephrine appropriately and with the proper dose and route of administration, as outlined in the UK Resuscitation Council guidelines on anaphylaxis[70]. Other reports have examined treatment patterns in the ED settings of civilian[71] and military hospitals[72] in the United States and observed that epinephrine injections were administered during acute anaphylaxis to 16% and 50% of patients, respectively, as recommended by consensus anaphylaxis guidelines. Retrospective analysis of a national reporting database on ED visits in the United States from 1993 to 2004 revealed 12.4 million allergy-related visits to the ED, approximately 1% of all ED visits based on *International Classification of Diseases, Ninth Revision, Clinical Modification *coding. Anaphylaxis coding was rare (0.01% of all ED visits), although epinephrine was administered in 50% of those coded with anaphylaxis. Epinephrine administration documented in patients with acute allergic conditions was infrequent (11%), and the trend of use declined over the period of interest from 19% to 7% (*P *= 0.04)[73].

Primary care physicians have demonstrated similar knowledge gaps in their knowledge pertaining to anaphylaxis. For example, a questionnaire based on the clinical scenario of a child with peanut-induced anaphylaxis was used in a random sample of 468 pediatricians in the United States[74]. About half (56%) agreed that the scenario represented anaphylaxis and that treatment with epinephrine was indicated. Most (81%) correctly chose to discharge the child home with self-injectable epinephrine and either to refer to an allergist or to recommend further diagnostic testing (86%). Similar surveys have been done in other countries (several studies are cited in Pongracic and Kim[75]).

Studies have also demonstrated that many health care professionals are uncertain about how to use an epinephrine autoinjector and thus cannot properly instruct their patients[76,77]. Available resources may help physicians develop treatment plans and resolve any therapeutic quandaries[17,18,65]. Examples of written action plans can be downloaded over the Internet (see Additional Educational Resources).

## Underutilization of Epinephrine by Patients, Parents, and Caregivers

Fatalities during witnessed anaphylaxis, most of which occur outside of a medical facility, usually result from delayed administration of epinephrine. In a retrospective review of 6 fatal and 7 nonfatal episodes of food-induced anaphylaxis in children and adolescents, all subjects who survived had received epinephrine before or within 5 minutes of developing severe respiratory symptoms. None of the subjects with fatal attacks received epinephrine before the onset of severe respiratory symptoms[20]. Analysis of data from a national case registry of fatal food anaphylaxis in the United States indicates that very few individuals (7/63) had epinephrine autoinjectors available at the time of fatal reaction[23,25]. Similarly, Pumphrey[21] determined that although epinephrine was administered in 62% of the fatal anaphylactic reactions in the United Kingdom that he reviewed, in only 14% was it given before cardiac arrest. In a follow-up analysis of 48 cases of fatal food anaphylaxis from 1999 to 2006, Pumphrey and Gowland[24] reported that 19 (40%) had received epinephrine autoinjectors, but more than one half of the fatalities occurred in patients whose previous clinical reactions had been so mild that, in the opinion of the investigators, it was unlikely that a physician would have prescribed a precautionary epinephrine syringe.

Multiple factors may contribute to the lack of available epinephrine for administration during anaphylaxis that occurs outside of a medical facility. An international survey conducted under the auspices of the World Allergy Organization determined that epinephrine autoinjectors were available in about half of surveyed countries, and that the cost of an autoinjector in some countries was equivalent to the monthly salary of an average citizen[78]. Of 39 countries, autoinjectors containing 0.15-mg and 0.3-mg doses were available in 17 (44%) and 22 (56%), respectively.

Adherence to an action plan to keep epinephrine available at all times and to inject it during anaphylaxis is another concern. Kemp and colleagues[79] determined in a follow-up survey of patients that 32 (47%) of 68 did not have the recommended epinephrine autoinjector with them when they again experienced anaphylaxis from a previously identified culprit. In contrast, 31 (91%) of 34 patients with idiopathic anaphylaxis (that is, no culprit could be identified) had epinephrine available at the time of a subsequent episode. Implementation of an educational protocol with emphasis on carrying epinephrine increased the frequency of adherence from 53% to 92% over the ensuing 10 years[80]. Other studies have similarly reported that 50% to 75% of patients prescribed epinephrine carry it with them, of whom 30% to 40% can demonstrate proper administration technique[81-84]. Still others carry epinephrine but choose not to use it during anaphylaxis[32,85-87] or prefer to seek emergency medical assistance[21].

Few studies thus far have examined management of anaphylaxis in school or day care settings. These are reviewed in detail elsewhere[75]. Protection of children at risk for anaphylaxis in school, day care, or other settings requires an interdisciplinary approach[9]. Several resources are available for help in the school or day care setting (see Additional Educational Resources).

## Precautions for the Patient at Risk for Anaphylaxis

Optimizing prevention (Table 3) is crucial because future anaphylaxis may be fatal despite appropriate management. An allergist-immunologist can provide comprehensive professional advice on these matters and should be consulted if he/she is not already involved in the anaphylaxis plan of care. All patients at risk for future anaphylaxis should carry at least 1 epinephrine syringe and know how to administer it.

**Table 3:** Preventive Measures to Reduce the Risk for Anaphylaxis

I. General measures
Obtain thorough history to diagnose life-threatening food or drug allergy
Identify cause of anaphylaxis and those individuals at risk for future attacks
Provide instruction on proper reading of food and medication labels, where appropriate
Avoidance of exposure to antigens and cross-reactive substances
Optimal management of asthma and coronary artery disease
Implement a waiting period of 20 to 30 min after injections of drugs or other biologic agents
In the physician's office, consider a waiting period of 2 h if a patient receives an oral medication he/she has never previously taken
II. Specific measures for high-risk patients
Individuals at high risk for anaphylaxis should carry self-injectable syringes of epinephrine at all times and receive instruction on proper use with placebo trainer
MedicAlert (MedicAlert Foundation, Turlock, Calif) or similar warning bracelets or chains
Substitute other agents for β-adrenergic blockers, angiotensin-converting enzyme inhibitors, tricyclic antidepressants, and monoamine oxidase inhibitors, whenever possible
Agents suspected of causing anaphylaxis should be given orally if possible; if the intravenous route is needed, a slow supervised rate of administration is required
Where appropriate, use specific preventive strategies, including pharmacological prophylaxis, short-term challenge and desensitization, and long-term desensitization

Modified from Kemp[88].

## Conclusions

Based on available evidence, the benefit of using appropriate doses of intramuscular epinephrine in anaphylaxis far exceeds the risk (evidence category IV). Consensus opinion and anecdotal evidence recommend epinephrine administration "sooner rather than later," that is, when the initial signs and symptoms of anaphylaxis occur, regardless of their severity, because fatalities in anaphylaxis usually result from delayed or inadequate administration of epinephrine. Experts may differ on how they define the clinical threshold by which they define and treat anaphylaxis. However, they have no disagreement whatsoever that appropriate doses of intramuscular epinephrine should be administered rapidly once that threshold is reached. There is no absolute contra-indication to epinephrine administration in anaphylaxis, and all subsequent therapeutic interventions depend on the initial response to epinephrine. Development of toxicity or inadequate response to epinephrine injections indicates that additional therapeutic modalities are necessary. All individuals at increased risk of anaphylaxis should have an anaphylaxis action plan and carry epinephrine autoinjectors for self-administration. Such individuals (and their caregivers, as appropriate) should be assessed regularly for adherence with these recommendations and for the ability to demonstrate proper epinephrine administration technique with a placebo device.

## Additional Educational Resources on Anaphylaxis

### Web Sites

World Allergy Organization (http://www.worldallergy.org)

Resuscitation Council (http://www.resus.org.uk/siteindx.htm)

American Academy of Allergy, Asthma, and Immunology (AAAAI) (http://www.aaaai.org)

American College of Allergy, Asthma, and Immunology (http://www.acaai.org)

Joint Council of Allergy, Asthma, and Immunology (http://www.jcaai.org)

Food Allergy and Anaphylaxis Network (FAAN) (http://www.foodallergy.org)

Allergy UK (http://www.allergyuk.org)

Anaphylaxis Canada (http://www.anaphylaxis.org)

The Web sites of other national and regional allergy/immunology organizations also provide useful perspectives.

### Action Plans for Health Care Professionals

AAAAI (http://www.aaaai.org/members/resources/anaphylaxis_toolkit/action_plan.pdf)

Spanish language versions of the following AAAAI anaphylaxis materials are now available: *The AAAAI Anaphylaxis Emergency Action Plan, Killer Allergy *information page, *AAAAI Anaphylaxis Tips to Remember *brochure, and *AAAAI Anaphylaxis Easy Reader *page.

FAAN (English language version: http://www.foodallergy.org/actionplan.pdf; Spanish language version: http://www.foodallergy.org/spanishaction.pdf)

### School, Child Care, or Camp Settings

New South Wales Department of Health, Anaphylaxis Guidelines for Schools (http://www.health.nsw.gov.au/pubs/a/pdf/anaphylaxis.pdf)

FAAN (http://www.foodallergy.org/school/SchoolGuidelines.pdf)

### Information for Patients and Their Families

Anaphylaxis Campaign (http://www.anaphylaxis.org.uk)

FAAN (http://www.foodallergy.org)

Global Allergy and Anaphylaxis Links (http://www.worldallergy.org/links.shtml)
